# Functions and Regulation of *HAM* Family Genes in Meristems During Gametophyte and Sporophyte Generations

**DOI:** 10.1111/pce.15286

**Published:** 2024-11-18

**Authors:** Yuan Geng, Chong Xie, Cankui Zhang, Xing Liu, Yun Zhou

**Affiliations:** ^1^ Department of Botany and Plant Pathology Purdue University West Lafayette Indiana USA; ^2^ Purdue Center for Plant Biology Purdue University West Lafayette Indiana USA; ^3^ Department of Agronomy Purdue University West Lafayette Indiana USA; ^4^ Department of Biochemistry Purdue University West Lafayette Indiana USA

**Keywords:** gametophyte, GRAS‐domain, meristem, microRNA

## Abstract

A fascinating feature of land plants is their ability to continually initiate new tissues and organs throughout their lifespan, driven by a pool of pluripotent stem cells located in meristems. In seed plants, various types of meristems are initiated and maintained during the sporophyte generation, while their gametophytes lack meristems and rely on sporophyte tissues for growth. In contrast, seed‐free vascular plants, such as ferns, develop meristems during both the sporophyte and gametophyte generations, allowing for the independent growth of both generations. Recent findings have highlighted both conserved and lineage‐specific roles of the HAIRY MERISTEM (HAM) family of GRAS‐domain transcriptional regulators in various meristems throughout the land plant lifecycle. Here, we review and discuss how *HAM* genes maintain meristem indeterminacy in both sporophytes and gametophytes, with a focus on studies performed in two model species: the flowering plant *Arabidopsis thaliana* and the fern *Ceratopteris richardii*. Additionally, we summarize the crucial and tightly regulated functions of the microRNA171 (miR171)‐HAM regulatory modules, which define HAM spatial patterns and activities during meristem development across various meristem identities in land plants.

## Summary statement

Meristems sustain undifferentiated stem cells that drive continuous organ formation and shape the plant body. This review highlights the crucial roles of the HAIRY MERISTEM (HAM) family of GRAS‐domain transcriptional regulators in maintaining meristem indeterminacy across both the sporophyte and gametophyte generations. It also outlines the conserved regulatory module that defines HAM expression patterns in different types of meristems.

## 1 Introduction

Meristems in land plants consist of groups of undifferentiated stem cells capable of continuous division. They share conserved functions, maintaining their undifferentiated state while continually producing daughter cells that eventually differentiate into various tissues and organs (Heidstra and Sabatini 2014; Kean‐Galeno, Lopez‐Arredondo, and Herrera‐Estrella 2024; Meyerowitz 1997). Land plants undergo an alternation of generations, comprising the sexual gametophyte phase and the asexual sporophyte phase (Bowman 2022; Jill Harrison 2017). They develop different types of meristems at various phases of their lifecycle (Bowman 2022; Jill Harrison 2017). In seed plants, meristems are exclusively initiated and maintained during the sporophyte generation, such as shoot apical meristems (SAMs), which are responsible for producing above‐ground tissues (Gaillochet and Lohmann 2015; Heidstra and Sabatini 2014; Meyerowitz 1997). Gametophytes of seed plants grow dependently on their sporophyte tissues and lack any meristems or stem cells (Li and Ma 2002; McCormick 2004; Yadegari, 2004). Unlike seed plants, ferns—vascular plants that propagate by spores instead of seeds—develop meristems during both the sporophyte and gametophyte generations (Imaichi 2013; Plackett, Di Stilio, and Langdale 2015). Apical meristems in fern sporophytes have structures and morphology different from their counterparts in seed plants but share similar functions (Plackett, Di Stilio, and Langdale 2015). Additionally, during the gametophyte phase, ferns develop multicellular meristems, which are responsible for producing sex organs and sustaining the indeterminate growth of gametophytes until fertilization (Banks 1999; Imaichi 2013; Plackett, Di Stilio, and Langdale 2015; Wu et al. 2023; Wu et al. 2021; Wu et al. 2022).

Understanding how meristems remain undifferenitated during plant growth and development has been a long‐standing question in the field. Over the last 20 years, several key regulators and associated pathways have been isolated and analyzed, primarily in models of flowering plants (angiosperms) (Gaillochet and Lohmann 2015; Han, Liu, and Zhou 2020b; Kitagawa and Jackson 2019; Lindsay, Swentowsky, and Jackson 2024). Among them, the *HAIRY MERISTEM* (*HAM*) gene was initially identified and characterized in Petunia. It was named after the mutant phenotype exhibiting ectopic formation of hair‐like structures (trichomes) on shoot meristems (Stuurman, Jäggi, and Kuhlemeier 2002), suggesting a role for HAM in maintaining meristems in an undifferentiated state. After then, molecular genetic studies in several other angiosperm species demonstrated the conserved and essential role of the HAM family GRAS‐domain transcription factors in meristem maintenance (Engstrom et al. 2011; Geng et al. 2021b; Schulze et al. 2010; Zhou et al. 2015; Zhou et al. 2018). In this review, we focus on recent advances in understanding the functions of HAM in various meristems throughout the plant lifecycle and how *HAM* activity is restricted by the conserved mircoRNA171 (miR171) family (Geng et al. 2021b; Geng et al. 2024; Geng, Yan, and Zhou 2022; Geng and Zhou 2021a, 2021b; Han et al. 2020a; Han, Liu, and Zhou 2020b). Our discussion here is primarily based on studies in meristems from two model systems: the flowering plant *Arabidopsis thaliana* (hereafter referred to as Arabidopsis) and the fern model *Ceratopteris richardii* (hereafter referred to as Ceratopteris). We also summarize the *HAM* gene family and the miR171‐HAM regulatory modules in land plants, and discuss future perspectives and new directions in this field.

## 2 *HAM* gene family in Land Plants

HAM family homologs are widely present across many lineages of land plants, including bryophytes, lycophytes, ferns, gymnosperms, and angiosperms (Engstrom et al. 2011; Geng et al. 2021b). This suggests that the origin of the *HAM* gene family likely predates the divergence of land plants (Geng et al. 2021b). Comprehensive phylogenetic analysis has demonstrated that in many non‐angiosperm lineages, such as mosses, lycophytes, ferns, and gymnosperms, the *HAM* family is maintained with low copy numbers, typically consisting of only one or two members in each species. However, the HAM family members have significantly expanded in angiosperm lineages (Engstrom et al. 2011; Geng et al. 2021b). In angiosperms, HAM homologs are categorized into two distinct groups, Type I and Type II, likely derived from a whole‐genome duplication (WGD) event in a common ancestor of angiosperms (Albert et al. 2013; Geng et al. 2021b). Type II HAM members are retained in all examined angiosperm species, while Type I HAM members appear to have been independently lost in several orders of monocots (Geng et al. 2021b). Additionally, the *HAM* gene family has undergone relatively recent duplication events in several angiosperm lineages, leading to further expansions of this family. For example, there are only two *HAM* homologs—one Type I (*AmHAM1*) and one Type II (*AmHAM2*)—in *Amborella trichopoda*, which is the sole living sister species to all other angiosperms and shows no evidence of recent genome duplications (Albert et al. 2013). In contrast, up to nine HAM homologs have been identified in the *Musa acuminata* (banana) genome, which have undergone three rounds of lineage specific‐WGDs (D'Hont et al. 2012).

## 3 HAM Regulates Shoot Meristem Development During the Sporophyte Phase in Angiosperms

**Figure 1:**
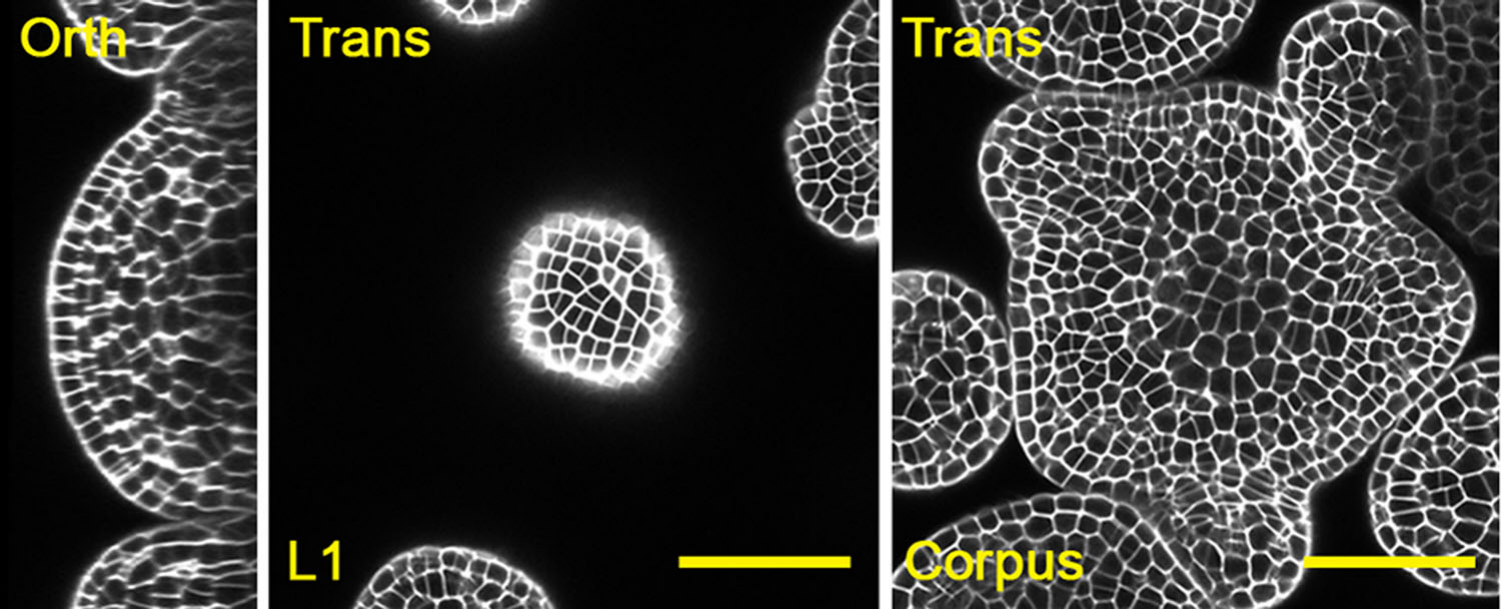
Confocal images of the Arabidopsis shoot apical meristem (SAM). Panels from left to right: an orthogonal view of an Arabidopsis SAM, a transverse section view of the L1 layer, and a transverse section view of the corpus/L3 layer in the same SAM. Scale bars: 50 µm. Gray: cell wall stain.

An Arabidopsis SAM consists of three clonally distinct layers (Figure 1). The outermost layer (L1) gives rise to the epidermis, while the underlying layer (L2) gives rise to the sub‐epidermis. The entire area beneath the L2 is the corpus, or L3 layer, where cells contribute to forming inner corpus tissues (Figure 1). Based on distinct cell fates and behaviors, the SAM can also be divided into different functional zones. Notably, a constant number of stem cells are maintained undifferentiated in the central zone (CZ) through a WUSCHEL (WUS)‐CLAVATA (CLV) feedback loop (Fletcher 2018; Schoof et al. 2000; Somssich et al. 2016).

Arabidopsis has four HAM members: three Type II (HAM1, HAM2, and HAM3) and one Type I (HAM4) (Engstrom et al. 2011; Geng et al. 2021b; Han et al. 2020a). Type II HAMs play roles in maintaining the WUS‐CLV3 feedback loop and sustaining the indeterminacy of shoot stem cell niches (Geng et al. 2021b; Han et al. 2020a; Han, Liu, and Zhou 2020b; Schulze et al. 2010; Zhou et al. 2015; Zhou et al. 2018). Specifically, HAM1 and HAM2 are expressed in the deeper layers of the SAM and function as interacting cofactors of WUS to regulate downstream targets and promote stem cell proliferation (Zhou et al. 2015). In the upper layers of the SAM, where *CLV3* is highly expressed, HAM1 and HAM2 are largely absent (Zhou et al. 2018). In the *ham123* mutant, the *CLV3* expression shifts from the upper to the deeper layers of the SAM (Geng et al. 2021b; Han et al. 2020a; Schulze et al. 2010; Zhou et al. 2018). Experimental results and computational simulations (Gruel et al. 2018; Liu, Shpak, and Hong 2020; Zhou et al. 2018) support a working model in which HAM, along with WUS, determines the localization of *CLV3* expression in the SAM (Han, Liu, and Zhou 2020b; Zhou et al. 2018). WUS activates *CLV3* expression in the CZ where HAM is absent but is unable to do so in the deeper layers where HAM is present. In addition, during the formation of new axillary meristems, HAM forms a concentration gradient that shifts the *CLV3* expression from the inner to the upper layers (Zhou et al. 2018). In the *ham123* mutant, *CLV3* remains in the inner layers, aligning with defects in axillary bud development (Engstrom et al. 2011; Geng et al. 2021b; Schulze et al. 2010; Wang et al. 2010; Zhou et al. 2018).

In several other angiosperm species, Type II *HAM* genes share conserved functions in maintaining SAMs and promoting axillary meristem formation (David‐Schwartz et al. 2013; Hendelman et al. 2016; Schulze et al. 2010; Stuurman, Jäggi, and Kuhlemeier 2002; Wang et al. 2010; Zhou et al. 2015; Zhou et al. 2018). For example, a mutation in the *CaHAM* gene in pepper (*Capsicum annuum*) leads to early termination of SAMs and arrested axillary shoots, similar to the phenotype displayed by *ham* loss‐of‐function mutants in Petunia and Arabidopsis (David‐Schwartz et al. 2013). Consistently, in cross‐species complementation studies, Type II *HAM* members from a monocot (rice) and two dicot species (pepper and soybean) rescued the developmental defects of Arabidopsis *ham123* (Geng et al. 2021b). Notably, the ability to maintain meristem indeterminacy in Arabidopsis shoot meristems has also been preserved in both Type I and Type II HAM from Amborella (the sister species to all other angiosperms) and in HAM members from non‐angiosperms, such as the gymnosperm *Larix kaempferi*, the fern Ceratopteris, the lycophyte *Selaginella moellendorffii*, and the moss *Physcomitrium patens* (Geng et al. 2021b).

## 4 HAM Regulates Meristem Development During the Gametophyte Phase in Ferns

**Figure 2:**
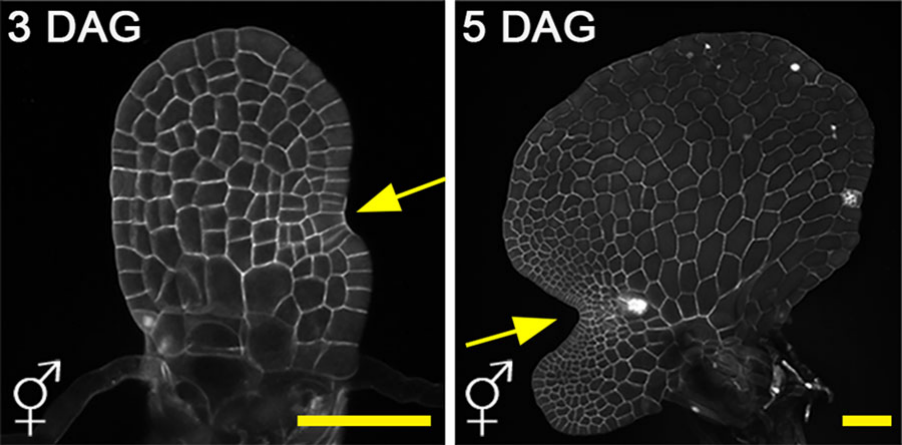
Initiation and establishment of multicellular meristems in Ceratopteris gametophytes. Confocal images of two hermaphroditic gametophytes at 3 and 5 days after germination (DAG). Yellow arrows indicate meristems at early (left) and late (right) developmental stages. Scale bars: 100 µm. Gray: cell wall stain.

Unlike seed plants, fern gametophytes are autotrophic, free‐living, and grow independently of their sporophytes. Similar to many fern taxa, the model fern Ceratopteris is homosporous, producing only one type of spore. During the haploid gametophyte phase, the genetically identical spores of Ceratopteris can develop into one of two sex types—male or hermaphrodite—depending on the influence of the pheromone antheridiogen (Banks 1999; Banks, Hickok, and Webb 1993; Hickok, Warne, and Slocum 1987). Antheridiogens from various fern taxa have been characterized, and so far, the identified antheridiogens from several fern species share structural similarities with gibberellic acids (Hickok, Warne, and Slocum 1987; Banks 1999; Banks, Hickok, and Webb 1993; Tanaka et al. 2014; Yamane 1998). In the presence of antheridiogen, a Ceratopteris spore develops into an ameristic male, lacking any meristem, with almost all the cells eventually differentiating into sperm‐producing antheridia (Hickok, Warne, and Slocum 1987; Banks 1999; Banks, Hickok, and Webb 1993). In contrast, when cultivated in isolation without exogenous antheridiogen, a Ceratopteris spore develops into a largely expanded, meristic hermaphrodite, characterized by a multicellular meristem, several egg‐producing archegonia, and a few sperm‐producing antheridia (Banks 1999; Hickok, Warne, and Slocum 1987) (Figure 2). The multicellular meristem is crucial for Ceratopteris hermaphrodite development, as it maintains continuous cell division activity within the meristem region, contributing to prothallus expansion. Additionally, the meristem constantly triggers adjacent cells to initiate and form archegonia, which bear eggs for fertilization (Banks 1999; Geng, Yan, and Zhou 2022). Interestingly, within a gametophyte population, once a hermaphrodite establishes itself and initiates the meristem, it begins producing and secreting antheridiogen into the environment (Hickok, Warne, and Slocum 1987; Banks 1999; Banks, Hickok, and Webb 1993). This pheromone triggers neighboring late‐germinating, sexually undetermined gametophytes to develop as males and promotes antheridium formation (Hickok, Warne, and Slocum 1987; Banks 1999; Banks, Hickok, and Webb 1993; Tanaka et al. 2014; Yamane 1998). Meanwhile, the hermaphrodite becomes insensitive to both endogenous and exogenous antheridiogen, preventing its conversion to a male in the presence of antheridiogen (Banks 1999; Banks, Hickok, and Webb 1993). This strategy, employed by many fern species including Ceratopteris, promotes outcrossing and enhances genetic diversity within populations. It also prevents all individuals in the same population from becoming males, thereby ensuring efficient sexual reproduction and species maintenance. While this phenomenon is intriguing, the underlying molecular mechanism had been a puzzle for more than three decades (Banks, Hickok, and Webb 1993; Hickok, Warne, and Slocum 1987).

**Figure 3:**
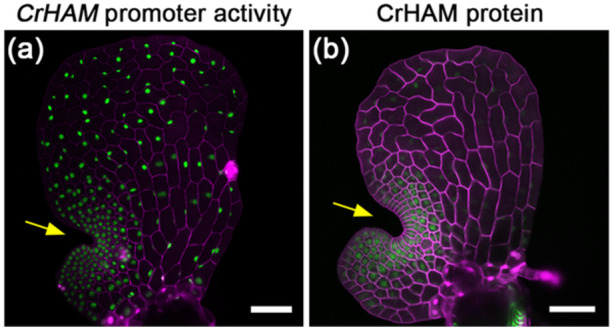
*CrHAM* expression patterns in Ceratopteris gametophytes. Confocal images of hermaphroditic gametophytes expressing the miR171‐insensitive transcriptional reporter *pCrHAM::H2B‐GFP* (a) and the miR171‐sensitive translational reporter *pCrHAM::YPET‐CrHAM* (b). (a) Merged channels of GFP (green) and cell wall stain (magenta). (b) Merged channels of YFP (green) and cell wall stain (magenta). Yellow arrows indicate meristems. Scale bars: 100 µm.

Fortunately, publicly available genomic and transcriptomic resources (Geng et al. 2021a; Marchant et al. 2022), the development of transient and stable transformation systems in Ceratopteris (Bui et al. 2015; Plackett et al. 2014), and the establishment of noninvasive confocal live‐imaging systems (Geng, Yan, and Zhou 2022), have greatly facilitated gene functional studies in Ceratopteris (Bui et al. 2017; Plackett, Di Stilio, and Langdale 2015; Plackett et al. 2018). A recent study on the Ceratopteris HAM family gene (hereafter referred to as *CrHAM*) has helped solve the puzzle and uncover the role of the multicellular meristem in the sex type specification (Geng et al. 2024). Specifically, on the same days after germination (DAG), *CrHAM* exhibits significantly higher expression in Ceratopteris hermaphrodites compared to males (Geng et al. 2024). The CrHAM protein preferentially accumulates in the multicellular meristem during its initiation and establishment in Ceratopteris hermaphrodites but is excluded from differentiated organs such as sperm‐producing antheridia in males. Once the meristem is fully established in hermaphrodites, CrHAM is restricted to the meristem (Geng et al. 2024) (Figure 3).

The *CrHAM* loss‐of‐function knockdown (KD) transgenic lines in Ceratopteris display developmental defects during the gametophyte phase and an increased male‐to‐hermaphrodite ratio, both of which are closely related to the identity and activity of multicellular meristems in hermaphrodites. Specifically, while *CrHAM* KD males are morphologically comparable to wild‐type (WT) males, *CrHAM* KD hermaphrodites exhibit reduced meristem size and disturbed meristem notch, with increased or even ectopic formation of antheridia (Geng et al. 2024). In the presence of antheridiogen, cells within or adjacent to the meristem of *CrHAM* KD gametophytes differentiate into antheridia, leading to the conversion of a significant number of hermaphrodites to males in the population (Geng et al. 2024). This finding is consistent with quantitative assays showing that *CrHAM* KD gametophytes increase sensitivity to exogenous antheridiogen (Geng et al. 2024). Additionally, CrHAM sustains meristem cell division and proliferation independent of antheridiogen. In the absence of exogenous antheridiogen, *CrHAM* KD hermaphrodites display reduced division activity, total cell number, and gametophyte size compared to WT hermaphrodites at the same DAG. These findings support a working model that CrHAM, specifically localized in meristems, sustains cell division and represses antheridiogen‐induced male differentiation programming, thereby maintaining meristem identity and promoting hermaphrodite development. Transcriptomic profiling of different sex types and genotypes at two developmental stages of gametophytes further demonstrates that CrHAM maintains meristem indeterminacy and hermaphrodite identity by integrating multiple conserved regulatory pathways, including transcriptional cascades, cell wall modification, auxin biosynthesis, and CLV peptide signals (Geng et al. 2024). Interestingly, during the gametophyte phase of Physcomitrium, another seed‐free model, *PpGRAS12* (a Physcomitrium *HAM* homolog) may also act as a positive regulator of meristem development, as overexpression of *PpGRAS12* leads to overproliferation of apical meristems on each gametophore (Beheshti et al. 2021). All these findings suggest a conserved role of HAM members in regulating meristems in both gametophytes and sporophytes.

## 5 microRNA171 (miR171): Conserved Regulators in Shaping HAM Patterns and Confining HAM Functions

microRNA171 (miRNA171) is a group of small noncoding RNAs whose precursors are encoded by *MICRORNA171* (*MIR171*) genes (Han and Zhou 2022; Llave et al. 2002b; Reinhart et al. 2002; Zhu et al. 2015). In Arabidopsis, all three Type II *HAM* genes (*HAM1‐3*) contain identical 21‐nt miR171 binding sites (5'‐GATATTGGCGCGGCTCAATCA‐3'), through which miR171 specifically recognizes and mediates the cleavage of *HAM1‐3* transcripts (Llave et al. 2002a; Llave et al. 2002b; Rhoades et al. 2002; Wang et al. 2010; Takanashi et al. 2018). Consistently, gain‐of‐function (such as ectopic expression) of *AtMIR171* in Arabidopsis results in reduced *HAM* levels and phenotypes resembling the *ham123* loss‐of‐function, including disorganized shoot meristem structures, aberrant *CLV3* expression domains, and arrested axillary branches (Wang et al. 2010; Zhou et al. 2018). In Arabidopsis SAMs, the *MIR171* genes are directly activated by the L1‐specific transcription factors ATML1 and its close homolog PDF2 (Han et al. 2020c). As a result, mature miR171 is synthesized exclusively in the epidermal layer (L1) and moves downward over a limited distance, leading to the cleavage of Type II *HAM* transcripts in the upper layers of SAMs (Han et al. 2020c). This creates a concentration gradient of miR171 from the epidermis to the inner layers, which defines an inverse concentration gradient of Type II HAM members along the apical‐basal axis in the SAMs (Han et al. 2020c). Notably, a miR171‐insensitive *HAM2* fluorescent transcriptional reporter is ubiquitously activated in all cell layers of SAMs (Han et al. 2020c). In contrast, the fluorescence signal of a miR171‐sensitive *HAM2* translational reporter is absent in the L1 but strong in the inner layers of SAMs (Han et al. 2020c).

Phylogenetic studies have revealed that the 21‐nt miR171 binding sites are highly conserved within the coding sequences of *HAM* members across non‐angiosperm lineages and in the majority of Type II *HAM* genes in angiosperms (Engstrom et al. 2011; Geng et al. 2021b). In contrast, in Type I *HAM* genes, the miR171 binding sites show diversification, with a few exceptions in species such as Amborella, *Nelumbo nucifera* and *Vitis vinifera* (Engstrom et al. 2011; Geng et al. 2021b). In parallel, *MIR171* family members are widespread across various land plant lineages, and mature miR171 sequences from different species display high levels of similarity (Cuperus, Fahlgren, and Carrington 2011; Geng et al. 2024; Han and Zhou 2022; Zhu et al. 2015). For instance, in 14 land plant species, including angiosperms and non‐angiosperms, miR171 sequences share 11 invariant nucleotides positions out of 21 (Geng et al. 2024). Additionally, overexpression of *MIR171* genes in tomato (*Solanum lycopersicum*) and rice (*Oryza sativa*) leads to the silencing of Type II *HAM* homologs and disrupts meristem development (Fan et al. 2015; Hendelman et al. 2016), providing evidence of a functional miR171‐HAM regulatory cascade across angiosperm lineages.

In the fern Ceratopteris genome, we recently identified two *CrMIR171* genes, *CrMIR171B* and *CrMIR171C* (Geng et al. 2024). Products of these two gene are predicted to form stem‐loop pri‐miRNA structures that are highly comparable to those produced by Arabidopsis *MIR171B* and *MIR171C*, respectively. The mature CrmiR171b and CrmiR171c also share nearly identical sequences with Arabidopsis miR171b/c (Geng et al. 2024). Consistently, CrmiR171b and CrmiR171c are highly complementary to the miR171‐binding site within the *CrHAM* transcript, which, surprisingly, remains identical to that in all three Arabidopsis Type II *HAM* genes (Geng et al. 2021b; Geng et al. 2024). More importantly, ectopic activation of *CrMIR171B* leads to a significant reduction of the *CrHAM* level in transgenic Ceratopteris gametophytes. These gametophytes display phenotypes similar to those observed in *CrHAM* loss‐of‐function transgenic gametophytes, such as the reduced expression of downstream targets, an increased male‐to‐hermaphrodite ratio in the population, reduced meristem size, and disturbed prothallus expansion in hermaphrodites (Geng et al. 2024).

**Figure 4:**
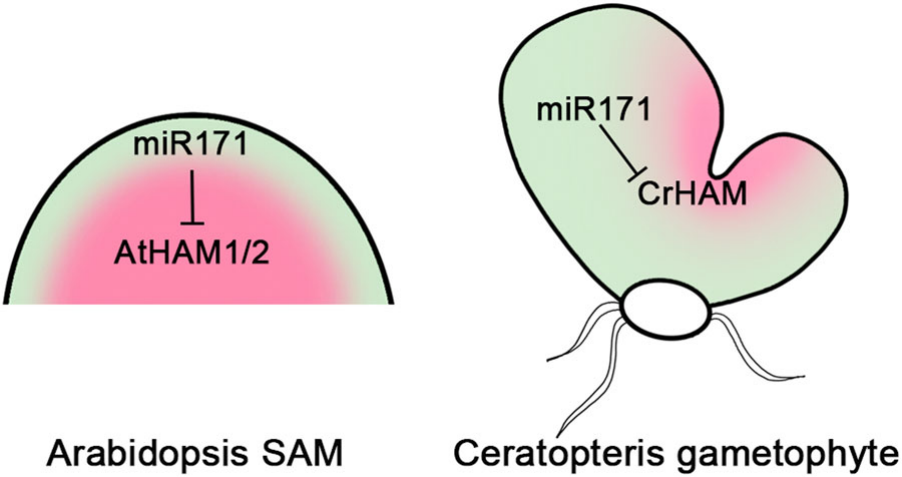
Diagrams illustrating HAM functions in the Arabidopsis SAM (sporophyte phase) and the Ceratopteris multicellular meristem (gametophyte phase). In both meristems, *HAM* family genes maintain meristem indeterminacy. The miR171‐HAM regulatory cascade defines the HAM expression domains in both meristems. Red indicates HAM protein localization patterns.

In a previous study, confocal imaging results demonstrated that the conserved miR171‐binding site in *CrHAM* can be recognized by Arabidopsis miR171, which is sufficient to establish an apical‐basal concentration gradient of YPET‐CrHAM in Arabidopsis SAMs when expressed under the control of the ubiquitously expressed Arabidopsis *HAM2* promoter (Geng et al. 2021b). During the gametophyte phase in Ceratopteris, the miR171‐insensitive *CrHAM* transcriptional reporter is ubiquitously expressed in the prothalli at different developmental stages (Geng et al. 2024; Geng, Yan, and Zhou 2022) (Figure 3a). In contrast, the miR171‐sensitive *CrHAM* translational reporter shows differential expression patterns, preferably accumulating in the meristems during initiation and proliferation, and eventually being restricted within the meristem once it is fully established in gametophytes (Geng et al. 2024) (Figure 3b). The direct comparison of these two reporters under the control of the same *CrHAM* promoter reveals miR171 activity in Ceratopteris gametophytes (Geng et al. 2024) (Figure 3a,b). Interestingly, in the moss Physcomitrium, a miR171‐insensitive reporter of *PpGRAS12* also shows an expression pattern different from that of a miR171‐sensitive reporter (Beheshti et al. 2021). Altogether, these results suggest the miR171 family likely plays a conserved role in shaping HAM patterns and restricting HAM activity in land plants (Figure 4).

## 6 Future Perspectives

Over the past several years, significant advances have been made in understanding the role of the *HAM* family in meristem development and its spatial and temporal regulation by miR171 throughout various stages of the plant life cycle. However, several major questions and challenges remain to be resolved.

First of all, current data suggest that while HAM family members maintain conserved or even interchangeable functions as meristem regulators in various lineages (Geng et al. 2021b; Han et al. 2020a), they appear to involve different downstream genes and signaling pathways in distinct meristems (Geng et al. 2024; Zhou et al. 2015; Zhou et al. 2018). It will be worthwhile and exciting to determine the molecular mechanisms by which HAM gates through different targets and pathways to achieve the same goal—keeping meristems from differentiation—in different types of meristems across species (Figure 4). In addition, similar to previous works focusing on Arabidopsis SAMs (Han et al. 2020c; Zhou et al. 2018), developing new computational models to predict and simulate complex signaling circuits centered on miR171‐HAM in various meristem identities, including those in the haploid gametophyte phase of Ceratopteris, will provide comprehensive and quantitative insights into the evolution and functions of this conserved regulatory module in land plants. Moreover, studies in both Arabidopsis and Ceratopteris have demonstrated that HAM patterns in meristems are primarily determined by miR171 (Figure 4) (Geng et al. 2024; Han et al. 2020c). Understanding how *MIR171* genes and mature miR171 are dynamically regulated throughout life cycles and among different plant lineages deserves future attention. More broadly, considering the essential roles of HAM in keeping meristem undifferentiated, fine‐tuning *MIR171* activity through modifications in their transcriptional regulators or cis‐regulatory elements within their promoters could efficiently alter meristem size and activity. This, in turn, could positively impact shoot architectures and biomass production during the sporophyte phase, as well as sexual reproduction during the gametophyte phase.

## Conflicts of Interest

The authors declare no conflicts of interest.

## Data Availability

Data sharing not applicable to this article as no datasets were generated or analysed during the current study.
